# Repair of a Chronic DeBakey Type II Aortic Dissection With Aortic Valve Insufficiency

**DOI:** 10.1016/j.atssr.2022.12.012

**Published:** 2023-01-12

**Authors:** Timothy W. James, Joshua N. Baker, Vinay Badhwar, J. Scott Rankin

**Affiliations:** 1 Department of Cardiac Surgery, St. Joseph’s Medical Center, Tacoma, Washington; 2 Department of Cardiac Surgery, Missouri Baptist Hospital, St Louis, Missouri; 3 Department of Cardiovascular and Thoracic Surgery, West Virginia University, Morgantown, West Virginia

## Abstract

Ascending aortic aneurysms with aortic insufficiency caused by chronic DeBakey type II dissections have been difficult to repair. A 70-year-old woman presented with heart failure, severe aortic insufficiency, and a large ascending aortic aneurysm. She had a trileaflet valve and a healed chronic proximal intimal flap. A 19-mm ring was sutured beneath the annulus, a 28-mm Dacron graft was anastomosed to zone 2, and the bovine innominate artery was connected to the graft. After bypass, the leaflets moved well, residual leak was negligible, and mean pressure gradient was 7 mm Hg. The patient recovered uneventfully.

Aneurysms localized to the ascending aorta can be associated with multiple pathologic processes. The most common are atherosclerosis, bicuspid disease, and connective tissue disorders. Chronic DeBakey type II dissections involving only the ascending aorta^1^ are less common but constitute an important cause. Aortic insufficiency (AI) can be produced by annular distortion or dilation and can be difficult to repair with existing techniques. A video case report is presented of an elderly woman who had aortic valve repair by geometric ring annuloplasty along with reconstruction of a large ascending aortic aneurysm.

A 70-year-old woman presented with heart failure from severe AI associated with an 8-cm ascending aortic aneurysm. On echocardiography, she had a trileaflet aortic valve with relatively normal cusps, a normal aortic root, and a well-functioning left ventricle (Video). The AI was central and severe. At the operation, a healed chronic proximal intimal flap was evident at the sinotubular junction, with thin dissection tissue beyond (Figure). Leaflet quality and coaptation height were good, but the noduli were thickened and noncompliant. The annulus was barely 21 mm, and the leaflets sized to a 19-mm annuloplasty ring. With use of 6 horizontal mattress sutures of 3-0 braided polyester supported with fine polyester pledgets, a 19-mm trileaflet annuloplasty ring^2^ was implanted beneath the annulus, and preliminary thinning of the thickened noduli was performed. A 28-mm Dacron graft fit the aorta, and under circulatory arrest with antegrade cerebral perfusion, the dissected aortic wall was excised to the left subclavian takeoff at zone 2. The distal anastomosis was performed, a bovine innominate artery was anastomosed to a graft side arm, and perfusion was resumed through the graft. Final nodular thinning was performed, and then the leaflets coapted well in the midline (Video; Figure). The proximal anastomosis was completed as air was evacuated. After bypass, the leaflets moved well with good coaptation height and a trivial residual leak. The vena contracta was <2 mm, and the pressure half-time was 650 milliseconds. The postrepair mean pressure gradient was 7 mm Hg. The patient recovered uneventfully and returned to full activity. Now beyond 1-year postoperatively, the patient is leading a normal life.

**Figure:**
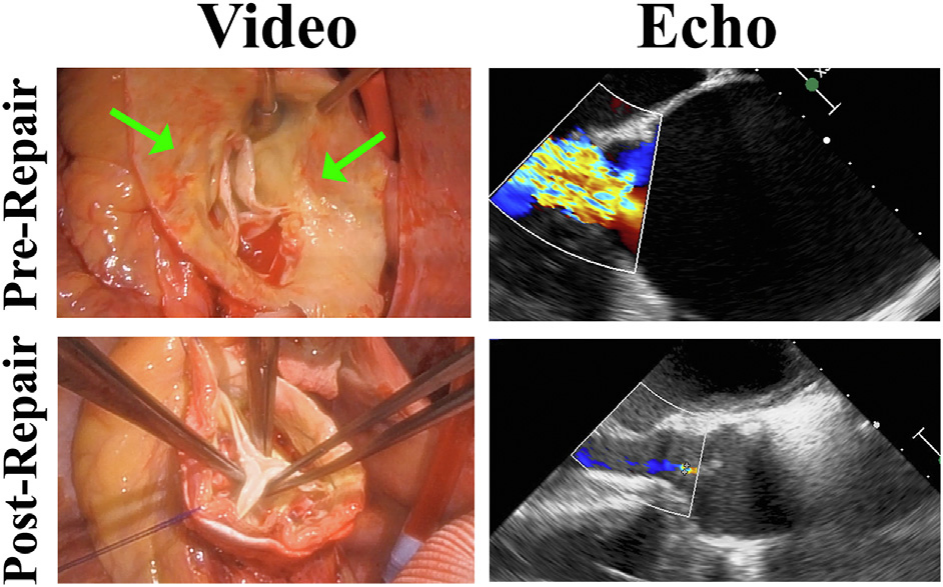
Insufficient aortic valve due to chronic proximal dissection (arrows indicate healed intimal flaps), repaired with geometric ring annuloplasty and ascending aortic graft.

## Comment

Chronic DeBakey type II dissections can be manifested as large ascending aortic aneurysms with severe AI in elderly patients. By aortic ring annuloplasty, valve repair is simple, especially if leaflet anatomy is preserved and the AI is caused primarily by annular distortion. The 19-mm geometric ring is especially useful in patients with small valves, who might exhibit higher gradients with tissue valve replacement. Implantation of a 19-mm ring requires only 6 stitches and is expeditious. Restoration of normal annular geometry often eliminates the AI, as in this case, but leaflet reconstruction can be performed when necessary. Midterm to long-term results with this approach have been excellent for both trileaflet^3^^,^^4^ and bicuspid^5^ valves, demonstrating low operative mortalities and 7- to 8-year survival free of reoperation or valve-related complications approximating 80% to 90%. Even in the elderly population, valve-related complications are low,^6^ so repair might be a serious consideration in older patients such as this. If sinuses are involved with the dissection, remodeling selective sinus replacement can be performed,^7^ and patients with adherent or prolapsed leaflets can be reconstructed easily with living autologous ascending aortic patches.^8^ Thus, autologous repair of AI associated with ascending aortic aneurysm due to chronic type II aortic dissection is now a simple and highly effective procedure.

## References

[1] DeBakey M.E., Cooley M.A., Creech O. (1955). Surgical considerations of dissecting aneurysm of the aorta. Ann Surg.

[2] Rankin J.S., Wei L.M., Downey R.S. (2021). Aortic valve repair using geometric ring annuloplasty. Oper Tech Thorac Cardiovasc Surg.

[3] Baker J.N., Klokocovnik T., Miceli A. (2022). Minimally invasive aortic valve repair using geometric ring annuloplasty. J Card Surg.

[4] Rankin J.S., Mazzitelli D., Fischlein T.J. (2018). Geometric ring annuloplasty for aortic valve repair during aortic aneurysm surgery: two-year clinical trial results. Innovations (Phila).

[5] Gerdisch M.W., Reece T.B., Emerson D. (2022). Early results of geometric ring annuloplasty for bicuspid aortic valve repair during aortic aneurysm surgery. JTCVS Tech.

[6] Aicher D., Fries R., Rodionycheva S., Schmidt K., Langer F., Schafers H.J. (2010). Aortic valve repair leads to a low incidence of valve-related complications. Eur J Cardiothorac Surg.

[7] Perez-Tamayo A., Baker J.N., Gerdisch M.W. (January 2022). Reconstruction of complex proximal aortic dissections with aortic insufficiency using aortic ring annuloplasty. https://www.ctsnet.org/article/reconstruction-complex-proximal-aortic-dissections-aortic-insufficiency-using-aortic-ring.

[8] Myers J.L., Clark J.B., James T.W. (2023). Use of aortic wall patches as leaflet replacement material during aortic valve repair. J Thorac Cardiovasc Surg.

